# QTL Analysis of Stem Elongation and Flowering Time in Lettuce Using Genotyping-by-Sequencing

**DOI:** 10.3390/genes12060947

**Published:** 2021-06-21

**Authors:** O New Lee, Keita Fukushima, Han Yong Park, Saneyuki Kawabata

**Affiliations:** 1 Department of Bio-Industrial and Bioresource, Sejong University, Neungdong-ro 209, Gwangjin-gu, Seoul 05006, Korea; hypark@sejong.ac.kr; 2 Graduate School of Agricultural & Life Sciences, The University of Tokyo, Yayoi 1-1-1, Bunkyo-ku, Tokyo 113-8657, Japan; pn11cjkf01ft@gmail.com (K.F.); skawabata@g.ecc.u-tokyo.ac.jp (S.K.)

**Keywords:** lettuce, QTLs, genotyping-by-sequencing, bolting, stem elongation, flowering time, leaf number

## Abstract

Lettuce plants tend to undergo floral initiation by elongation of flower stalks (bolting) under high-temperature and long-day conditions, which is a serious problem for summer lettuce production. Our objective was to generate a high-density genetic map using SNPs obtained from genotyping-by-sequencing (GBS) analysis of F5 recombinant inbred lines (RILs) and to map QTLs involved in stem growth and flowering time in lettuce. A set of 127 intra-specific RIL mapping populations derived from a cross between two varieties, green and red leaf lettuce, were used to identify QTLs related to the number of days from sowing to bolting (DTB), to flowering of the first flower (DTF), to seed-setting of the first flower (DTS), and the total number of leaves (LN), plant height (PH), and total number of branches of main inflorescence (BN) for two consecutive years. Of the 15 QTLs detected, one that controls DTB, DTF, DTS, LN, and PH detected on LG 7, and another QTL that controls DTF, DTS, and PH detected on LG 1. Analysis of the genomic sequence corresponding to the QTL detected on LG 7 led to the identification of 22 putative candidate genes. A consistent QTL related to bolting and flowering time, and corresponding candidate genes has been reported. This study will be valuable in revealing the genetic basis of stem growth and flowering time in lettuce.

## 1. Introduction

Lettuce (*Lactuca sativa* L.) is one of the most important leafy vegetables cultivated worldwide and consumed throughout the year [1,2]. It belongs to the Asteraceae family, and is a self-fertilizing diploid plant with 2n = 2x = 18 chromosomes and an estimated 2.5 Gb genome size [1,3]. It is beneficial to human health as it contains compounds such as vitamins C and E, polyphenols, fibers, tocopherols, and lutein [4]. Lettuce plants tend to undergo floral initiation at temperatures higher than 20 °C and under long photoperiods [5,6]. Bolting refers to the rapid elongation of the inflorescence axis and stem internode [7]. The differentiation of the inflorescence meristem and the division of the intercalary meristem are both responsible for bolting. The inflorescence meristem appears as a floral transition by which flowering plants switch from vegetative growth to reproductive growth [8]. The vegetative shoots can be discerned from the flower stalks by histomorphological changes; DNA replicating cells are distributed more uniformly and are frequently associated with floral initiation [9]. The log-linear relationship between stem length and stem diameter deviates from linearity as the stem elongates exponentially with time after flower initiation [6]. Bolting causes leaves to become bitter and limits crop marketability. In iceberg-type lettuce, flower stalks elongate in a circular manner inside the head due to tight head formation [10]. Thus, the resultant damage in crop quality has become a serious economic problem in the summer production of lettuce.

Bolting coincides with the development of flower buds due to the coordinated effects of developmental and environmental factors [6,7]. Lettuce bolting consists of the following two developmental processes: flower initiation and stem internode elongation. Therefore, the differentiation of the inflorescence meristem and division of the intercalary meristem are both responsible for bolting. The shoot apical meristem differentiates into the floral meristem, which then develops various floral organs during the bolting period. In situ hybridization using histone H4 gene expression detected the earliest event of floral initiation at the shoot apical meristem, which occurred three days prior to stem internode elongation in lettuce [11]. Premature bolting and early flowering are damaging to the propagation of lettuce, and adversely affect cultivation management, the crop yield, and seed production. The flowering time is closely associated with stem internode elongation in rosette plants, which is an important trait in breeding programs for bolting resistance [10]. Previous studies determined these traits to be regulated by multiple genes that were inherited as qualitative characteristics [10,12,13]. The molecular regulation remained unclear, although several genes were reported to be involved in the inflorescence development and bolting processes in lettuce, such as FLOWERING LOCUS T (*LsFT*) and SUPPRESSOR OF OVEREXPRESSION OF CONSTANS1 (*LsSOC1*) [1].

Despite the agricultural and biological interest in lettuce, knowledge of its genetics and genome is very limited. Quantitative trait loci (QTL) analysis has been performed on lettuce with increased focus on root architecture [11], seed and seedling traits [12,13], post-harvest shelf life [14], resistance to downy mildew [15], lettuce drop [16], and physiological disorders [17]. The genetic architecture of lettuce was investigated using a recombinant inbred line population from a cross between *Lactuca sativa* “Salinas” and its wild relative *L. serriola* [18], in which the alleles causing a delay in flowering time were detected on linkage group (LG) 7. Flowering time, bolting, and stem elongation are essential traits for lettuce breeding; shortening of the vegetative phase and elongation of the internodal region decreases leaf production, the genetic mechanism of which remains unclear.

A high-density linkage map is a prerequisite for successful QTL identification [19]. Genotyping-by-sequencing (GBS) analysis has been widely applied in various plant species including in apple, chickpea, barley, maize, rice, wheat, and soybean [12,20,21,22,23,24,25]. The GBS approach allows for sequencing, discovery, and genotyping of thousands of single nucleotide polymorphisms (SNPs) in a single-step broad scale in a cost-effective manner [26]. SNPs have been proven to be ubiquitous in high numbers, with uniform distribution, biallelic nature, and of high heritability [27,28]. The GBS protocol requires a relatively small amount of starting DNA (100–200 ng) and restriction enzymes to reduce genome complexity. Recombinant inbred lines (RILs) are adequate for genetic mapping because they can improve the mapping resolution with a high number of recombination, be replicated by seeds, and facilitate a better estimation of the QTL effects.

The purpose of this study was to (1) discover large-scale SNPs using GBS; (2) generate a high-resolution linkage map with simultaneous genotyping of an intraspecific mapping population of lettuce; (3) identify the QTLs responsible for varietal differences in bolting time in lettuce; and (4) predict putative candidate genes for the major QTLs detected.

## 2. Material and Methods

### 2.1. Mapping Population

Two non-heading type lettuce lines of *L. sativa* were used to produce an intraspecific RIL mapping population. One hundred and twenty-seven F5 RILs were derived from a cross between two varieties of lettuce: green leaf lettuce with early stem elongation (*Lactuca sativa* L. cv. Chimasanchu; Sakata Seed Co., Yokohama, Japan; female parent), and red leaf lettuce with late stem elongation (*Lactuca sativa* L. cv. Banchu Red Fire; Sakata Seed Co., Yokohama, Japan; male parent). The resultant F1 population was advanced using the single-seed descent method to obtain the F5 population, which was then used for two phenotypic evaluations under greenhouse conditions and for GBS analysis.

### 2.2. Phenotyping of the RIL Population

For phenotypic evaluation, 127 F5 RILs, along with their parents, were grown in a greenhouse under natural daylight at the University of Tokyo, Japan (35.72° N, 139.76° E). The average daily temperatures in 2013 and 2014 were 22.8 ± 5.2 °C (minimum and maximum temperatures: 7.4 and 33.2 °C) and 22.2 ± 4.7 °C (minimum and maximum temperatures: 9.0 and 31.1 °C), respectively. The monthly average light durations in 2013 and 2014 were 170.8 ± 43.5 and 176.5 ± 39.0 h, respectively. Five seedlings from each of the RILs were grown in 1 L plastic pots (14.5 cm diameter) containing a mixture of commercial growth mediums “Red ball soil” (Plantation Iwamoto, Ibaraki, Japan) and “Metro-Mix 360” (Sun Gro Horticulture, Washington, DC, USA) in the ratio 2:1. During cultivation, a 1/500-fold diluted Hyponex solution (Hyponex, Osaka, Japan) was administered to the plants on a weekly basis. Cultivation was conducted in a greenhouse from April to October, in both 2013 and 2014. To evaluate the stem growth and flowering time of the 127 F5 RILs, we measured six stem growth and flowering-related traits: the number of days from sowing to bolting (DTB; the first flower bud was observed outside the plant as the stem elongated), the number of days from sowing to flowering of the first flower (DTF), the number of days from sowing to seed setting of the first flower (DTS), total number of leaves (LN), plant height (PH) when the first flower fully opens, and the total number of branches of the main inflorescence (BN). PH (cm) was measured using relevant rulers. Means, standard errors, and correlation coefficients were calculated for each trait of the parents as well as the 127 F5 population using SPSS 12.0 KO for Windows (SPSS, Chicago, IL, USA), with a significance level of 5%.

### 2.3. Genotyping-by-Sequencing

DNA libraries for GBS were constructed according to previously described protocols [26,29], with minor modifications. The serial restriction digestion of DNA with *Ape*kI and *Mse*I at 37 °C for 2 h and again at 75 °C for 2 h was followed by ligation with adapters. The adapters included a set of 96 different barcode-containing adapters for tagging individual samples and a common adapter for all samples. Ligation was performed using 200 cohesive end units of T4 DNA ligase (New England Biolabs, MA, USA) at 22 °C for 2 h, and then the ligase was inactivated by incubation at 65 °C for 20 min. The sets of 95 ligations were pooled into one sample and purified using the QIAquick PCR Purification Kit (Qiagen, Chatsworth, CA, USA). The pooled ligations (5 µL) were amplified in 50 µL reaction by multiplex PCR using AccuPower Pfu PCR Premix (Bioneer, Daejeon, South Korea) and 25 pmol of each primer. PCR cycles consisted of an initial step at 98 °C for 5 min, followed by a total of 18 cycles of 98 °C for 10 s, 65 °C for 5 s, and 72 °C for 5 s, with a final extension step at 72 °C for 5 min. The PCR products were purified using the QIAquick PCR Purification Kit (Qiagen, Chatsworth, CA, USA), and the distribution of fragment sizes was evaluated using BioAnalyzer 2100 (Agilent Technologies, Santa Clara, CA, USA). The GBS libraries were sequenced using Illumina NextSeq500 (Illumina, San Diego, CA, USA) and had a length of 150 bp single-end reads for a total of 129 DNA samples.

Sequenced reads were demultiplexed with “process_radtags” module in Stacks tool [30]. Chromosome level genome data from the Lettuce Genome Resource (http://lgr.genomecenter.ucdavis.edu (accessed on 1 April 2018)) were used as reference for lettuce (Reyes-Chin-Wo et al., 2017). After demultiplexing, single-end sequence reads were mapped to the lettuce reference genome using Bowtie2 [31]. For calling variants, we used the Genome Analysis Toolkit (GATK) and Picard tools (McKenna et al., 2010). We conducted local realignment of reads to correct any misalignments caused by the presence of insertions and deletions, using GATK “RealignerTargetCreator” and “IndelRealigner” sequence data processing tools. Subsequently, GATK “HaplotypeCaller” and “SelectVariants” instructions were used to call variants. Variants were further filtered using GATK “FilterVariants” instructions and VCFtools.

### 2.4. Linkage Map Construction and QTL Mapping

Custom code was used to transform the VCF formatted SNP data into an input format using the R/Qtl package. Markers with distorted segregation ratios were filtered using the chi-square test with a *p*-value threshold of 0.05. R package “ASMap” was used to construct linkage map with a *p*-value threshold of 1 × 10^−6^ and objective function as the maximum likelihood [32]. The composite interval mapping (CIM) function in the R/Qtl package was used for QTL mapping, along with the Kosambi mapping function.

### 2.5. Whole Genome Resequencing and Annotation

The DNA library was prepared using the Illumina TruSeq Nano DNA HT Kit according to the manufacturer’s protocol. Initially, the extracted DNA was fragmented into indexed shotgun paired-end libraries (~550 bp inserts) using Covaris M220 (Woburn, MA, USA). Subsequently, the fragments of DNA were end-repaired, 3′ end adenylated, and adapter ligated before they were subjected to size selection and amplification. Quality control was further carried out on the resulting DNA library using a 2100 Bioanalyzer (Agilent Technologies, Palo Alto, CA, USA), which analyzes the size distribution of the DNA and detects contamination. Finally, paired-end sequencing was performed using the Illumina Novaseq system, which produced ~89 Gbp output for all samples.

Quality control was performed using fastQC [33] and Trimmomatic [34] to remove the low-quality bases of reads and adaptor sequences. The high-quality reads obtained after quality control were mapped to *Lactuca sativa V8* genome [3] using Bowtie2 (http://bowtie-bio.sourceforge.net/bowtie2/index.shtml/ (accessed on 1 April 2018)) with default settings. Picard tool (http://broadinstitute.github.io/picard/ (accessed on 1 April 2018)) was used to sort the reads mapped to the reference genome, to remove PCR duplicates, and to fix mate-pair information. The reference and bam files were indexed using SAMtools [35]. Before obtaining high-quality variants, we conducted local realignment to correct misalignments caused by insertions and deletions using GATK [36]. Finally, “UnifiedGenotyper”, “SelectVariants”, and “filterVariant” arguments implemented in GATK were used to call variants, select SNPs, and filter SNPS, respectively, by using the following options: a Phred-scaled quality score < 30, quality score by allele depth < 5, MQ0 (total count across all samples of mapping quality zero reads) > 4, and a Phred-scaled *p*-value using Fisher’s exact test > 200, to reduce false positive calls. VCFtools 0.1.15 [37] was used to select biallelic SNPs and then filtered with the option—minDP 5.

Annotation of SNPs and INDELs was performed using SnpEff 4.3v [38] along with a database constructed using *Lactuca sativa V8* [3] genome and gene files. Genomic and coding sequences were substituted with variants detected in the coding regions for each sample.

## 3. Results

### 3.1. Trait Variation

**Figure 1:**
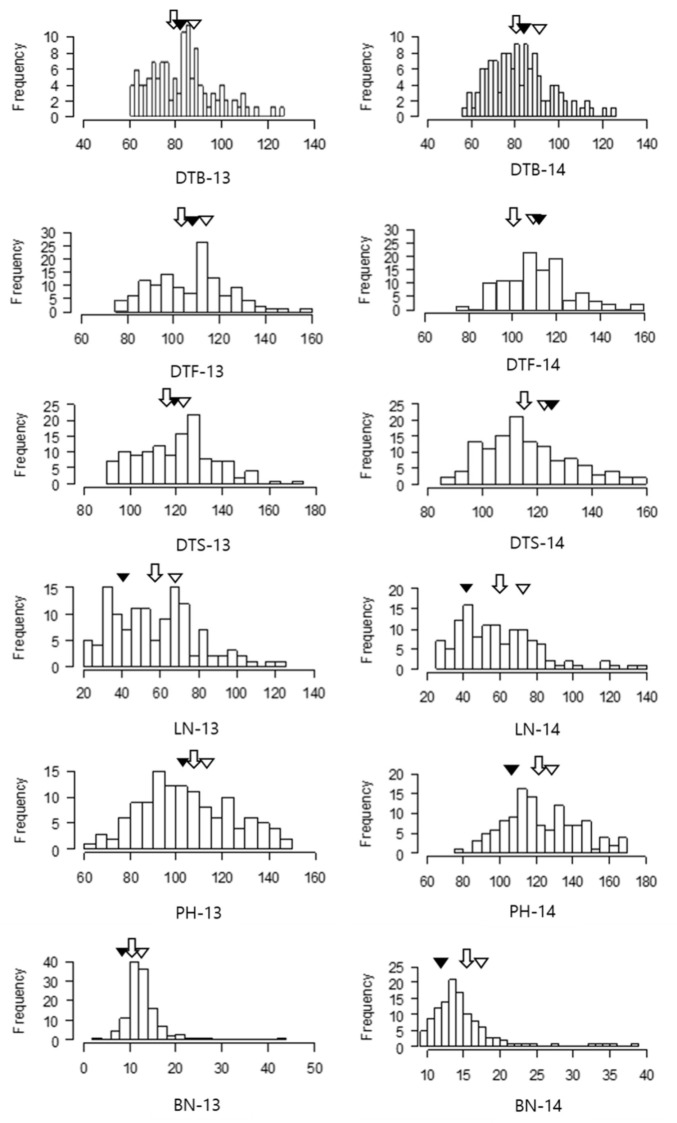
Frequency distribution for each trait in the F5 RIL population. The suffix “-13” refers to the spring trial conducted in 2013, while “-14” refers to the spring trial in 2014. Means for the parental and F5 RIL population are shown by arrows. (▽) *Lactuca sativa* L. cv. Chimasanchu (*n* = 10); (▼) *Lactuca sativa* L. cv. Banchu Red Fire (*n* = 10); (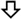) F5 (*n* = 127).

All analyzed traits showed a continuous unimodal distribution among the RILs (Figure 1, Table 1). The Chimasanchu cultivar (CS) showed higher values for DTB, LN, PH, and BN, when compared to those of the Banchu Red Fire cultivar (RF) in both years. CS plants bolted later compared to that did RF (1.2 days and 4.3 days in 2013 and 2014, respectively). However, the DTF and the DTS differed in 2013 and 2014; DTF and DTS of CS showed higher values in 2013 and RF showed higher DTF and DTS values in 2014. For the LN, PH, and BN traits, the average values of RILs were intermediate between those of CS and RF. CS plants differentiated more leaves (LN; 27.6 and 32.7 in 2013 and 2014, respectively), showed increased rapid elongation of main stem (PH; 10.8 cm and 18.6 cm in 2013 and 2014, respectively), and developed more branches of main inflorescence (BN; 3.2 and 7.58 in 2013 and 2014, respectively) when compared to those of the RF plants.

### 3.2. Correlations between Traits

The correlation coefficients between the six traits were calculated for the RILs over two years (Table 2). All six traits (DTB, DTF, DTS, LN, PH, and BN) were significantly and positively correlated with each other (*p* < 0.05). The Pearson’s correlation coefficients(r) among DTB, DTF, and DTS were very high (*r* > 0.95), and these traits were strongly correlated with each other. LN and PH also showed high correlations with DTB, DTF, and DTS in both years. LN showed a significant correlation with PH, with a correlation coefficient of approximately 0.5. BN showed a significant correlation with DTB, DTF, DTS, and LN, but showed a relatively low correlation with PH (*r* < 0.25).

### 3.3. SNP Discovery by GBS and Construction of Genetic Map

To develop genome-wide SNPs from lettuce using the GBS approach, two restriction enzymes (*Ape*KI and *Mse*I) were used to digest genomic DNA of the 127 RILs and their two parents. A total of 210.3 million cleaned reads with a total of 31.7 Gb were generated. Of these, 195.62 million high-quality filtered reads successfully passed the QC steps; the remaining reads were eliminated due to lack of proper layout of the barcode and restriction sites. The number of reads obtained varied from 0.5 to 2.7 million among the 127 RILs, with an average of 1.40 million reads for each line. The obtained sequences were filtered and used for SNP identification. Of the cleaned reads, 92.7% were successfully mapped to the reference sequence of the *Lactuca sativa V8* genome. Finally, 164,895 SNPs were identified from the SNP calling using GATK quality filtering and biallelic filtering. After excluding SNPs that were monomorphic in the RIL population, more than 5% of missing data, less than 5% of minor allele frequency (MAF), and less than 5% of mean depth, 1845 fine set of SNPs remained. A high-density genetic map of nine linkage groups was constructed with 1503 SNPs (Table 3, Supplementary Figure S1, Supplementary Table S1). The map covered a total of 1773.5 cM genetic distance ranging from 111.6 cM (LG 6) to 277.8 cM (LG 4), with an average of 197.1 cM for each linkage group and 1.20 cM between adjacent markers. The number of filtered SNPs and their frequencies varied across linkage groups. The largest number of SNPs was detected on LG 2 (229 SNPs) and lowest on LG 6 (77 SNPs). Based on the estimated genome size, the average genome-wide ratio of physical to genetic distance was 1231 kb per cM, equivalent to one SNP marker per 1453 kb.

### 3.4. QTL Analysis

**Figure 2:**
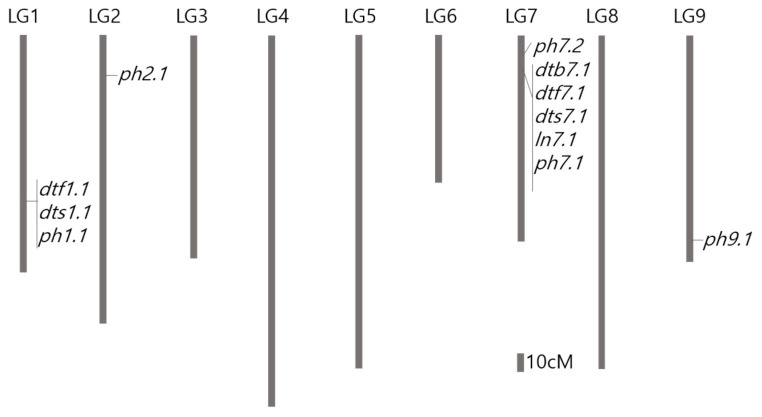
Chromosomal location of QTLs for the number of days from sowing to bolting (DTB), the number of days from sowing to flowering of the first flower (DTF), the number of days from sowing to seed setting of the first flower (DTS), total number of leaves (LN), and plant height (PH) of *Lactuca sativa* L. cv. Chimasanchu (P1), *Lactuca sativa* L. cv. Banchu Red Fire (P2), and F5 RIL population. QTLs with significant LOD scores determined by performing 1000 permutations (*p* < 0.05) are shown.

Fifteen QTLs were detected for the five traits related to stem elongation and flowering time in lettuce (Table 4). One region was identified as harboring several QTLs that regulated stem elongation and flowering time; a total of nine QTLs were identified in the same region of LG 7 (20.73 cM) (Figure 2). *dtb7.1*, *dtf7.1*, and *dts7.1* were detected in the same region of LG 7, and CS alleles resulted in increases in DTB, DTF, and DTS. In addition, three QTLs were mapped on LG 1 (127.65 cM), and the other three QTLs for PH were separately located on LG 2 and LG 7. The LOD peaks for BN did not reach the threshold determined using 1000 permutations. One DTB QTL (*dtb7.1*) was detected on LG 7, which explained 15% and 12% of the phenotypic variation of the trait in 2013 and 2014, respectively. Two QTLs for DTF (*dtf1.1* and *dtf7.1*) were detected on LGs 1 and 7, respectively, with *dtf1.1* accounting for 8% of the DTF phenotypic variance in 2013. The RF alleles in *dtf1.1* increased the DTF. *dtf7.1* accounted for 53% and 11% of the phenotypic variation in 2013 and 2014, respectively, and the presence of CS alleles increased the number of days to flowering. Two DTS QTLs (*dts1.1* and *dts7.1*) were detected in the same region as the DTF QTLs on LG 1 and LG 7. *dts1.1* was only detected in 2013, while *dts7.1* was detected in both years. The RF alleles in *dts1.1* increased the DTS, while the CS alleles in *dts7.1,* increased the DTS. One LN QTL on LG 7 (*ln7.1*) was detected in the same region as DTB, DTF, and DTS, and explained 10% and 6%

Of the phenotypic variation in 2013 and 2014, respectively. The presence of CS alleles in *ln7.1* increased the leaf number. Analysis of plant height at the time of seed setting revealed five significant QTLs. Four QTLs (*ph1.1*, *ph2.1*, *ph7.1*, and *ph9.1*) were detected in 2013, and one QTL (*ph7.2*) was detected only in 2014. Two PH QTLs on LGs 1 and 2 were contributed by the RF alleles. *ph7.1* was detected in the same region as other QTLs on LG 7 (*dtb7.1*, *dtf7.1*, *dts7.1*, and *ln7.1*), with the largest contribution to the phenotypic variation (37%). The presence of CS alleles in the QTLs increased plant height for the two PH QTLs (*ph7.1* and *ph9.1*).

### 3.5. Candidate Gene Prediction for QTLs Controlling Stem Elongation and Bolting

Here, we attempted to search for the candidate gene in the region of Chr7 where DTB, DTF, DTS, LN, and PH QTLs formed clusters and were detected for two consecutive years. One common QTL among DTB, DTF, DTS, LN, and PH on LG 7 was selected for candidate gene analysis. To identify potential candidate genes underlying QTLs, whole-genome resequencing analysis and annotation of the two parents were conducted in this study. A total of 203,257,298 and 173,641,982 reads for CS and RF, respectively, were generated after the two parental samples were resequenced. The average Q30 ratio was 91.78%, and average resequencing depths were 17.15× and 24.93× for the two parents, CS and RF, respectively. The average alignment rate was 89.38%, and the genome coverage was 89.55%. A total of 3,912,108 SNPs and 375,650 INDELs were identified between the two parents, on comparison with the “*Lactuca sativa* V8” reference genome. Variants from the coding and intergenic regions were distinguished. Corresponding to the QTLs on LG 7, it was preliminarily located in a 6.17 Mb candidate region between 159,881,847 bp to 166,054,789 bp of chromosomes. The genetic positions of the SNPs were in accordance with their physical positions. This region was subjected to structural and functional annotation, and 22 candidate genes were identified (Table 5). Among them, several genes were expected to function in the developmental process of lettuce.

## 4. Discussion

Here, we performed genotyping-by-sequencing (GBS) analysis combined with quantitative trait loci (QTL) analyses for stem elongation, bolting, and flowering-time related traits using an intraspecific F5 RIL population derived from two lettuce cultivars (Table 3). The GBS approach is suitable for genetic analysis and marker development of lettuce [39,40]. In this study, we constructed a highly saturated linkage map using GBS analysis covering a total of 1773.5 cM with 1503 SNPs with the genetic distance ranging from 111.6 cM (LG 6) to 277.8 cM (LG 4). A new linkage map was recently constructed by ddRAD-seq analysis, a marker-based genotyping platforms, using genotypes of 4517 biallelic tag loci and similarly encompassing 1529.2 cM with the genetic distance ranging from 134.8 (LG7) to 213.8 cM (LG4). An intraspecific cross has the advantage of decreasing the genetic distortion and errors encountered in other reports using wide crosses to establish genetic maps [41]. However, the low level of genetic diversity from intraspecific cross hinders the acquisition of sufficient DNA markers and efficient QTL analyses. Marker-assisted selection (MAS), a complementary tool for conventional breeding, requires a large number of molecular markers to detect markers linked to the target trait. Large-scale single nucleotide polymorphisms (SNPs), such as GBS, RRL, and RAD, have been effectively developed with rapid progress in high-throughput sequencing analysis. GBS reduces genome complexity by using restriction enzymes to divide the genome into fragments, the ends of which are sequenced on short-read sequencing platforms [42]. It has been successfully applied in highly homozygous crops such as maize, rice, soybean, wheat, and barley to provide large numbers of SNP markers for association studies and genomics-assisted breeding [43,44,45,46]. Moreover, it does not require preliminary sequence information and all newly discovered markers originate from the population being genotyped, although sequenced regions are not evenly covered in all individuals within a population [47]. The initial protocol was developed using one restriction enzyme [44] and was subsequently modified to use two restriction enzymes (a common cutter and a rare cutter) to generate a uniform complexity reduction [45]. Here, we used the two-enzyme (*Ape*KI and *Mse*I) approach to reduce genome complexity by avoiding sequencing of repetitive regions, resulting in a suitable and uniform reduction in the complexity of the large genomes of lettuce.

A total of 15 QTLs were detected over two years, with ten and five QTLs detected in 2013 and 2014, respectively (Table 4). Four of these QTLs (*dtb7.1*, *dtf7.1*, *dts7.1*, and *ln7.1* on LG7) were detected in both years. In brief, *dtb7.1*, *dtf7.1*, *dts7.1*, *ln7.1*, and *ph7.1*, were mapped on the same regions 20.7 cM with the genetic intervals between 10.4 cM. These QTLs contributed to a considerable proportion of the phenotypic variation in the respective traits. However, the PVE values in the 2013 and 2014 experiments for *dtf7.1* and *dts7.1* differed. In a previous study, several flowering time QTLs on LG7 explained 11.2%, 30.23%, 39.6%, and 51.7% of the phenotypic variance in separate experiments that used the same mapping population (PI251246 × Salinas) [48]. It was suggested that the environment-sensitive QTLs represent genetic variations in the upstream signaling of the flowering time pathway, where environmental pressured are perceived and converted into molecular signals. There were significant correlations between these traits based on Pearson’s correlation analysis (Table 2). In a previous lettuce study, QTLs for shelf life, leaf biophysical, developmental, and growth-related traits were identified in the same region of LG 7 [14,18]. QTL and PCA results showed that the clustering on LG 7 (15.5‒22.4 cM) was the most important region for growth-related and earliness traits, including the proportion of stem leaves after 30 days, plant height after 60 days, days to first flower, days to first seed, and plant height at seed set in lettuce [18]. Hartman et al. [18] suggested that QTL clustering is caused by pleiotropic effects from a common major gene for flowering, because the peak values of five major QTLs co-localize within 9 cM. Park et al., (2020) conducted GWAS for bolting time using 441 lettuce accessions by GBS analysis [49]. In total, 146 SNPs spanning nine regions across the genome were significantly associated with bolting time, with an FDR cutoff of 0.05 (*p*-value < 3.6 × 10^−5^). Among the nine regions, five (two on chromosome 4, one on chromosome 7 and two on chromosome 8) overlapped with the results or crisphead lettuce, and the most significantly SNPs associated were located in the ~164 Mb region of chromosome 7 which is also where the QTLs related bolting clustered in this study. Although further studies are required, it is possible that a single gene is responsible for the pleiotropic effects on multiple developmental processes in lettuce, such as stem elongation, flowering time, and leaf differentiation. Another possible interpretation is that the multiple genes with different functions for controlling bolting, flowering, and seed setting are localized within a cluster (functional gene clusters) on LG7 due to the large interval size of the detected QTLs [50]. Clustering of QTLs where similar or adjacent loci controlled several related traits might account for the occurrence of QTL hotspots [51]. Clustering QTLs could result from physical linkage among multiple genes that were individually selected, or have been integrated in the crop genome due to linkage drag [52,53]. In Chinese cabbage, the co-localization of QTLs controlling different quantitative traits suggested a close genotypic correlation between leaf and heading traits, which might be governed by different closely located genes or by a single gene with pleiotropic effects [50,54]. A single gene with pleiotropic effects on multiple developmental processes has been reported in several plant species, including in oilseeds *B. napus* and *B. juncea* [55,56]. For example, a gene for earliness which also affected branching patterns, number of days from flowering to fruiting, and pod number was detected in dry beans [57]. Moreover, a major flowering gene was also found to be involved in germination in Arabidopsis [58]. Due to the availability of the whole genome sequence of lettuce, it became possible to identify potential candidate genes underlying QTLs on LG 7 (Table 5). Putative candidate genes that govern stem elongation and flowering-related traits were identified. For example, phytochrome C was detected in this region, where 13 variant SNPs were located within a 3403 region. It is an essential light receptor for photoperiodic flowering and is responsible for perceiving light signals [59]. In rice, it plays an important role in FR-mediated repression of flowering under long-day conditions [60]. In maize, it has been reported to regulate early flowering and plant height [61]. Thus, phytochrome C could be a plausible candidate gene for the control of stem elongation and flowering. The zw10 gene, which is a control point for the formation of the mitotic spindle, and is involved in vascular transport between the ER and Golgi apparatus in the interphase, has been identified in *A. thaliana* [62,63]. Several receptor-like protein kinases have also been previously reported to be involved in cell differentiation, plant growth and development, self-incompatibility, hormonal response pathways, and symbiont and pathogen recognition [64]. Nitrate reductase (NR) is a key enzyme in regulating nitrate assimilation, which has been found to influence nitrate uptake and reduction in plants [65,66]. In addition, pentatricopeptide repeat (PPR) protein was detected in this region, which was strongly linked to 18 significantly associated SNPs. PPR is known to be involved in organelle biogenesis and post-transcriptional control [67]. To further specify the candidate gene, we need to design markers based on the candidate region for the early bolting or flowering-time lines and validate the gene through transformation analysis.

Delayed bolting and flowering is preferred for vegetable production; however, over-late flowering is unfavorable for seed production [48]. The presence of CS alleles in *dtb7.1*, and *ln7.1* on LG7 increased the number of days to bolting and number of leaves. This region could be useful for breeding cultivars with late bolting and high productivity. Identifying the molecular mechanism of developmental processes in lettuce, i.e., bolting, flowering, leaf differentiation, and seed-setting, may benefit genetic studies and breeding.

## 5. Conclusions

We generated a high-density genetic map using several thousand SNPs obtained using GBS analysis of a new recombinant inbred line population, developed through single seed descent from the intraspecific cross between two *Lactuca sativa* cultivars. Consequently, we also investigated the genetic control of stem elongation and flowering-related traits by QTL analysis. One consistent major QTL for bolting, flowering time, seed setting, leaf number, and plant height that corresponded to *dtb7.1*, *dtf7.1*, *dts7.1*, *ln7.1*, and *ph7.1*, was identified. Each QTL explained between 6.1‒55.2% of the phenotypic variance. SNP markers closely linked to traits can be used to select the preferred genotypes. Associated markers will be useful in breeding programs to develop plants having resistance to bolting, flowering, and stem elongation.

## Figures and Tables

**Table 1:** Average (±SD) and statistical analysis of the number of days from sowing to bolting (DTB), the number of days from sowing to flowering of the first flower (DTF), the number of days from sowing to seed setting of the first flower (DTS), total number of leaves (LN), plant height (PH) when the first flower fully opened, and total number of branches of main inflorescence (BN) of *Lactuca sativa* L. cv. Chimasanchu (P1), *Lactuca sativa* L. cv. Banchu Red Fire (P2), and F5 RIL population.

Year	Trait	Chimasanchu (P1)	Banchu Red Fire (P2)	F5
2013	DTB	88.00 ± 6.24	86.80 ± 2.59	83.10 ± 14.43
	DTF	111.60 ± 4.98	109.20 ± 3.90	105.48 ± 15.62
	DTS	124.20 ± 4.55	122.80 ± 3.90	118.69 ± 15.97
	LN	67.80 ± 7.36	40.20 ± 9.36	55.45 ± 21.59
	PH	113.60 ± 4.04	102.80 ± 4.09	102.13 ± 19.14
	BN	13.60 ± 2.60	10.40 ± 1.95	12.64 ± 3.24
2014	DTB	90.89 ± 5.37	86.56 ± 3.05	82.13 ± 13.72
	DTF	111.10 ± 4.33	112.00 ± 5.61	103.23 ± 14.51
	DTS	122.67 ± 5.61	125.67 ± 2.80	116.13 ± 15.50
	LN	75.00 ± 8.98	42.33 ± 4.16	59.32 ± 22.90
	PH	124.88 ± 9.00	106.33 ± 4.73	111.48 ± 20.38
	BN	17.25 ± 2.66	9.67 ± 2.31	15.33 ± 5.13

**Table 2:** Correlation between the traits examined in 2013 and 2014 (DTB—the number of days from sowing to bolting; DTF—the number of days from sowing to flowering of the first flower; DTS—the number of days from sowing to seed setting of the first flower, LN—total number of leaves; PH—plant height when the first flower fully opened; and BN—total number of branches of main inflorescence of *Lactuca sativa* L. cv. Chimasanchu (P1), *Lactuca sativa* L. cv. Banchu Red Fire (P2), and F5 RIL population * and ** indicate significance at *p* < 0.05 and *p* < 0.01, respectively.

Trait	Year	DTB	DTF	DTS	LN	PH
DTF	2013	0.969 **				
	2014	0.983 **				
DTS	2013	0.962 **	0.989 **			
	2014	0.982 **	0.997 **			
LN	2013	0.859 **	0.903 **	0.896 **		
	2014	0.676 **	0.685 **	0.678 **		
PH	2013	0.336 **	0.35 **	0.346 **	0.526 **	
	2014	0.266 *	0.268 *	0.268 *	0.464 **	
BN	2013	0.551 **	0.634 **	0.624 **	0.648 **	0.219 *
	2014	0.334 **	0.37 **	0.372 **	0.431 **	0.225 *

**Table 3:** Summary statistics of the lettuce intraspecific genetic linkage map constructed using the F5 RIL population derived from *Lactuca sativa* cv. Chimasanchu × *L. sativa* cv. Banchu Red Fire.

Linkage Group	Total Number of Mapped Markers	Genetic Length (cM)	Physical Length (bp)	Average Interval between Two Markers	
				cM	bp
1	212	175.9	208,403,342	0.8	983,035
2	229	216.1	209,216,842	0.9	913,611
3	115	167.3	235,685,571	1.5	2,049,440
4	209	277.8	359,266,232	1.3	1,718,977
5	216	249.8	332,823,101	1.2	1,540,848
6	77	111.6	172,193,396	1.4	2,236,278
7	108	155.4	178,722,727	1.4	1,654,840
8	210	249.7	302,195,534	1.2	1,439,026
9	127	169.9	185,552,563	1.3	1,461,044

**Table 4:** Quantitative trait loci (QTLs), their positions (cM), logarithm of the odds (LOD), percentage of phenotypic variation (PVE), additive effects (Add.), and their physical position (bp) for bolting, stem elongation and flowering time related traits in a F5 RIL population developed from *L. sativa* cv. Chimasanchu × *L. sativa* cv. Banchu Red Fire.

Trait	QTL	LG	Interval (cM)	Position (cM) ^a^	2013			2014			Physical Interval (bp)	Physical Position (bp)
					LOD ^b^	PVE ^c^	Add ^d^	LOD ^b^	PVE ^c^	Add ^d^		
DTB	*dtb7.1*	7	18.59–29.02	20.73	17.37	15.24	10.36	16.52	11.97	9.76	159,857,676–166,243,410	164,472,862
DTF	*dtf1.1*	1	123.11–129.39	127.65	4.35	8.25	−4.85				36,628,878–41,340,346	36,628,914
	*dtf7.1*	7	18.59–29.02	20.73	18.42	53.09	11.83	14.07	11.43	10.24	159,857,676–166,243,410	164,472,862
DTS	*dts1.1*	1	123.11–129.39	127.65	4.36	7.63	−4.78				37,964,416–41,340,346	36,628,914
	*dts7.1*	7	18.59–29.02	20.73	19.07	55.20	12.37	14.15	11.90	11.13	159,857,676–166,243,410	164,472,862
LN	*ln7.1*	7	18.59–29.02	20.73	14.00	10.82	13.28	7.67	6.14	12.18	159,857,676–166,243,410	164,472,862
PH	*ph1.1*	1	110.62–117.50	113.29	8.18	19.42	−8.49				45,487,674–49,165,461	50,734,059
	*ph2.1*	2	57.79–68.84	66.00	4.87	11.92	−6.93				169,936,949–179,130,539	172,151,268
	*ph7.1*	7	18.59–29.02	20.73	14.48	37.28	11.78				159,857,676–166,243,410	164,472,930
	*ph7.2*	7	8.84–16.79	14.60				4.51	15.55	7.88	166,971,907–175,172,138	167,324,134
	*ph9.1*	9	152.30–167.82	164.78	5.03	13.44	7.76				10,161,237–30,276,076	10,321,730

^a^ Position on chromosome in cM. ^b^ log-likelihood; phenotypic effect. ^c^ Percentage of phenotypic variance explained. ^d^ Positive values indicate that the contributing alleles were from P1 whereas negative values indicate the contributing alleles were from P2.

**Table 5:** List of SNPs identified in the QTL region on chromosome 7 and their annotated candidate genes.

Gene	ID	Gene Description	Molecular Function	CDS_ID	bp	Ref. ^a^	Alt. ^b^
At5g59700	Y5597_ARATH	Probable receptor-like protein kinase	Protein kinase activity	Lsat_1_v5_gn_7_95041	159,881,847	T	C
ZW10	ZW10_ARATH	Centromere/kinetochore protein zw10 homolog	Cell division	Lsat_1_v5_gn_7_94920	159,937,103	T	C
Gene	NIA_CICIN	Nitrate reductase [NADH]	Nitrate assimilation	Lsat_1_v5_gn_7_94901	159,961,111	G	A
At4g26790	GDL66_ARATH	GDSL esterase/lipase	Hydrolase activity	Lsat_1_v5_gn_7_94800	160,295,038	G	T
wss2	YQ77_SCHPO	Ubiquitin and WLM domain-containing metalloprotease	DNA-binding proteins	Lsat_1_v5_gn_7_94721	160,394,234	A	C
KAS	KASM_ARATH	3-oxoacyl-[acyl-carrier-protein] synthase, mitochondrial	3-oxoacyl-[acyl-carrier-protein] synthase activity	Lsat_1_v5_gn_7_94701	160,398,658	C	T
TRZ2	RNZ2_ARATH	tRNase Z TRZ2, chloroplastic	3′-tRNA processing endoribonuclease activity	Lsat_1_v5_gn_7_94680	160,401,079	T	C
At1g04970	Y1049_ARATH	Putative BPI/LBP family protein	Lipopolysaccharide binding	Lsat_1_v5_gn_7_94640	160,447,537	T	A
GDI1	GDIR_ARATH	Rho GDP-dissociation inhibitor 1	Rho GDP-dissociation inhibitor activity	Lsat_1_v5_gn_7_95621	162,399,118	G	T
rpoB	RPOB_LACSA	DNA-directed RNA polymerase subunit β	DNA-directed 5′-3′ RNA polymerase activity	Lsat_1_v5_gn_7_95881	163,026,039	T	C
ESS2	ESS2_HUMAN	Splicing factor ESS-2 homolog	Pre-mRNA splicing	Lsat_1_v5_gn_7_96161	163,390,527	C	T
Dnajb5	DNJB5_MOUSE	DnaJ homolog subfamily B member 5	Chaperone binding	Lsat_1_v5_gn_7_95320	163,700,503	T	C
PCMP-H35	PP373_ARATH	Putative pentatricopeptide repeat-containing protein	Zinc ion binding	Lsat_1_v5_gn_7_96961	164,472,930	G	A
PHYC	PHYC_ORYSJ	Phytochrome C	Phosphorelay sensor kinase activity	Lsat_1_v5_gn_7_96941	164,640,464	A	G
GNT2	MGAT2_ARATH	α-1,6-mannosyl-glycoprotein 2-β-N-acetylglucosaminyltransferase	Catalytic activity ^i^	Lsat_1_v5_gn_7_96920	164,651,092	G	A
ABC1K7	AB1K7_ARATH	Protein activity of BC1 complex kinase 7	Resistance to oxidative stress	Lsat_1_v5_gn_7_96461	164,942,842	G	C
To50-2rc	TO50-2rc	Transferase activity	Transferase activity	Lsat_1_v5_gn_7_96441	164,951,268	C	T
SHH2	SHH2_ARATH	Protein SAWADEE homeodomain homolog 2	Chromatin binding	Lsat_1_v5_gn_7_97960	164,983,376	A	G
At3g07680	P24B2_ARATH	Transmembrane emp24 domain-containing protein p24beta2	Intracellular protein transport	Lsat_1_v5_gn_7_97980	165,012,377	A	G
RLP7	RLP7_ARATH	Receptor-like protein 7	Receptor	Lsat_1_v5_gn_7_97661	165,751,808	G	A
RLP6	RLP6_ARATH	Receptor-like protein 6	Receptor	Lsat_1_v5_gn_7_97581	165,785,946	C	G
accD	ACCD_LACSA	Acetyl-coenzyme A carboxylase carboxyl transferase subunit β, chloroplastic	Carboxylase activity	Lsat_1_v5_gn_7_98321	165,894,155	G	T

^a^ The base that is the same as in the reference genome, Lactuca sativa V8 genome. ^b^ The other base that is the different with the reference genome. “Ref” represents Chimasanchu, and “Alt” represents Banchu Red Fire.

## Data Availability

No new data were created or analyzed in this study. Data sharing is not applicable to this article.

## Supplementary Materials

The following are available online at https://www.mdpi.com/article/10.3390/genes12060947/s1, Figure S1: High-density intra-specific linkage map of lettuce using GBS markers, Table S1: Genotypes of the genetic map constructed using GBS SNP markers.
