# Supplementary material for: QTL Analysis of Stem Elongation and Flowering Time in Lettuce Using Genotyping-by-Sequencing
Source: Genes (Basel). 2021 Jun 21;12(6):947. doi: 10.3390/genes12060947 (PMC8234873; doi:10.3390/genes12060947)
Supplement: Supplementary file 1 [file genes-12-00947-s001.zip › FigureS1.pdf]

Supplemental Materials

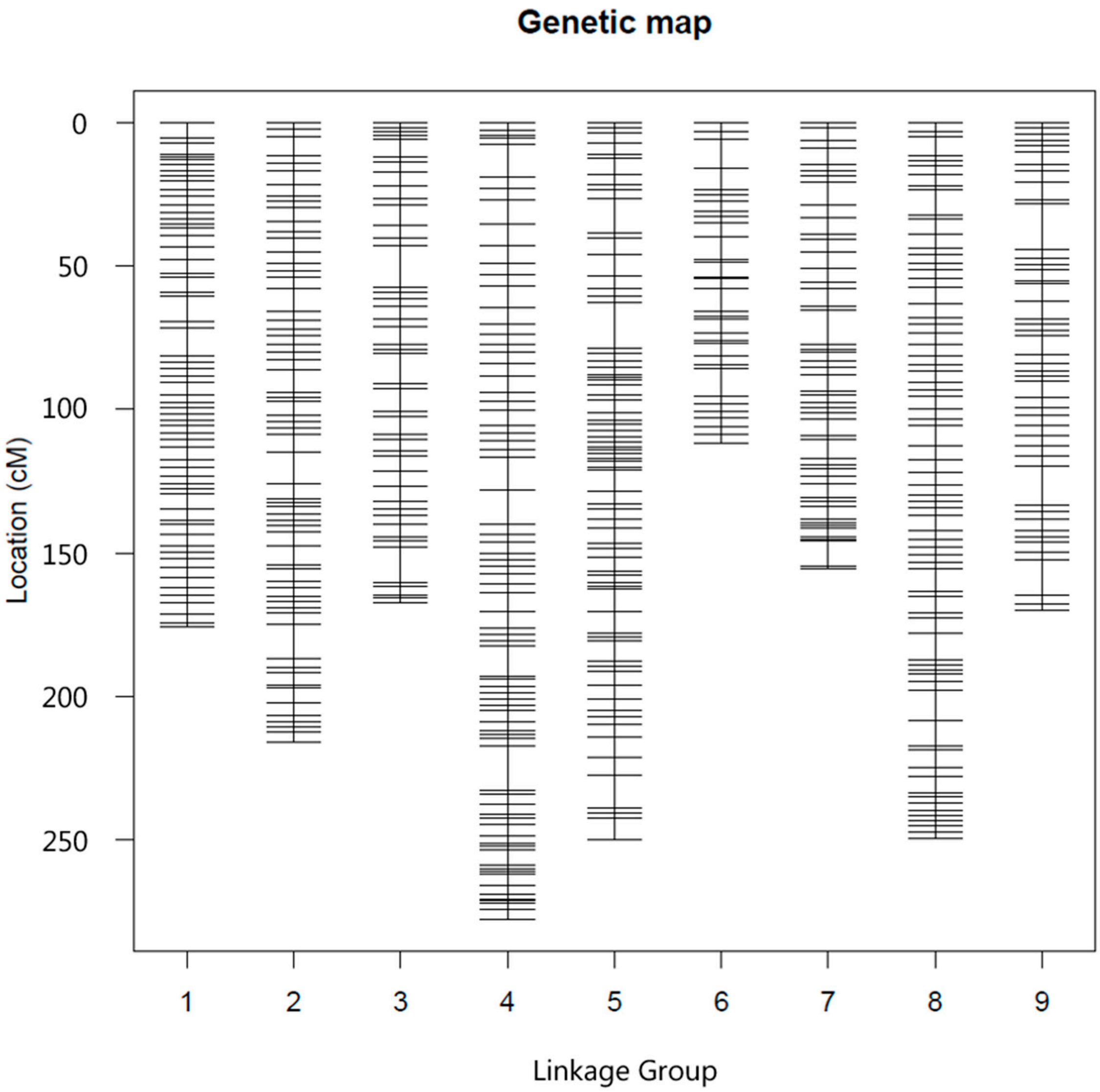

Figure S1. High-density intra-specific linkage map of lettuce using GBS markers.
